# High-flow nasal cannula oxygen therapy versus conventional oxygen therapy in patients with acute respiratory failure: a systematic review and meta-analysis of randomized controlled trials

**DOI:** 10.1186/s12890-017-0525-0

**Published:** 2017-12-13

**Authors:** Youfeng Zhu, Haiyan Yin, Rui Zhang, Jianrui Wei

**Affiliations:** 1 Department of Intensive Care Unit, Guangzhou Red Cross Hospital, Medical College, Jinan University, Tongfuzhong Road No. 396, Guangzhou, Guangdong province 510220 China; 2 Department of Cardiology, Guangzhou Red Cross Hospital, Medical College, Jinan University, Guangzhou, Guangdong province 510220 China

**Keywords:** High-flow nasal cannula, Mortality, Acute respiratory failure, Treatment

## Abstract

**Background:**

Acute respiratory failure (ARF) is a common and life-threatening medical emergency in patients admitted to the hospital. Currently, there is a lack of large-scale evidence on the use of high-flow nasal cannulas (HFNC) in patients with ARF. In this systematic review and meta-analysis, we evaluated whether there were differences between HFNC therapy and conventional oxygen therapy (COT) for treating patients with ARF.

**Methods:**

The EMBASE, Medline, and Wanfang databases and the Cochrane Library were searched. Two investigators independently collected the data and assessed the quality of each study. Randomized controlled trials that compared HFNC therapy with COT in patients with ARF were included. RevMan 5.3 was used to conduct the meta-analysis.

**Results:**

Four studies that involved 703 patients with ARF were included, with 371 patients in the HFNC group and 332 patients in the COT group. In the overall estimates, there were no significant differences between the HFNC and COT groups in the rates of escalation of respiratory support (RR, 0.68; 95% CI, 0.37, 1.27; z = 1.20, *P* = 0.23), intubation (RR, 0.74; 95% CI, 0.55, 1.00; z = 1.95, *P* = 0.05), mortality (RR, 0.82; 95% CI, 0.36, 1.88; z = 0.47, *P* = 0.64), or ICU transfer (RR, 1.09; 95% CI, 0.57, 2.09; z = 0.26, *P* = 0.79) during ARF treatment. However, the subgroup analysis showed that HFNC therapy may decrease the rate of escalation of respiratory support (RR, 0.71; 95% CI, 0.53, 0.97; z = 2.15, *P* = 0.03) and the intubation rate (RR, 0.71; 95% CI, 0.53, 0.97; z = 2.15, P = 0.03) when ARF patients were treated with HFNC therapy for ≥24 h compared with COT.

**Conclusions:**

HFNC therapy was similar to COT in ARF patients. The subgroup analysis showed that HFNC therapy may decrease the rate of escalation of respiratory support and the intubation rate when ARF patients were treated with HFNC for ≥24 h compared with COT. Further high-quality, large-scale studies are needed to confirm our results.

**Electronic supplementary material:**

The online version of this article (10.1186/s12890-017-0525-0) contains supplementary material, which is available to authorized users.

## Background

Acute respiratory failure (ARF) is a common and life-threatening medical emergency in patients admitted to hospitals [1]. It is caused by a variety of diseases, including heart failure, pneumonia, and exacerbations of chronic obstructive pulmonary disease. Many patients with ARF require oxygen therapy. The devices for oxygen therapy include unassisted oxygen delivery devices and assisted ventilation devices [2]. Unassisted oxygen therapy is also called conventional oxygen therapy (COT). It is the main supportive treatment administered to patients with ARF and is usually delivered with nasal prongs or facemasks. Assisted ventilation devices that are commonly used in hospitals include noninvasive ventilation (NIV, e.g., continuous positive airway pressure and biphasic positive airway pressure) and invasive mechanical ventilation (IMV). Previous studies have shown that avoiding IMV significantly decreases the risk of death [3, 4]. Therefore, choosing an optimal oxygen therapy device is very important for reducing the rates of IMV and mortality while also ensuring patients’ safety and comfort.

The effect of COT is limited. The maximal flow rate that these COT devices can deliver is typically only 15 L/min (except for the Venturi mask), which is far lower than the demands of patients with ARF. This discrepancy leads to a significant decrease in the fraction of inspired oxygen (FiO_2_) that ultimately reaches a patient’s lungs [5].

ARF patients admitted to the hospital may receive NIV. However, currently, the effects of NIV for these patients with respect to improvements in outcomes are conflicting and the use of NIV in hypoxemic ARF has recently been questioned [6–8]. Furthermore, NIV is not without limitations. The effect of NIV is highly dependent on a patient’s cooperation, which is also called patient-ventilator synchrony. Additionally, there are many factors that affect the comfort of patients undergoing NIV that may lead to NIV failure, such as the interface, the amount of air leaks, the ventilator settings, pressurization and triggering performances [9]. Moreover, NIV is associated with gastric distension, which may further reduce the functional residual capacity and is poorly tolerated in some patients. [10, 11] Therefore, IMV may still be needed [11].

The high-flow nasal cannula (HFNC) is a recently developed oxygen therapy device in adult patients that can deliver a humidified and heated mixture of air and oxygen at a very high flow rate. It can provide a maximal flow rate of up to 60 l per minute with an FiO_2_ of 100% [5]. The use of an HFNC has been demonstrated to generate positive airway pressure at end-expiration, ameliorate oxygenation and dyspnea, reduce the work of breathing and the respiratory rate, and be more comfortable for patients [5, 12–19]. These benefits are attributed to the mechanisms of HFNCs, including their ability to more adequately meet the peak flow of inspiration, flush the anatomical dead space, and deliver warm and humidified gas, thereby promoting mucociliary function [20, 21].

The use of HFNCs has become increasing popular in the treatment of many diseases and conditions, such as post-extubation, pre-intubation, sleep-related hypoventilation, cardiac surgery, and heart failure, and as an alternative to NIV [20]. However, currently, whether ARF patients benefit from this therapy is unclear and there is a lack of large-scale evidence on the use of HFNCs in patients with ARF. Some studies have shown that HFNC therapy is associated with an improved respiratory state or mortality rate in patients with ARF [2, 22]. However, other studies have not found significant differences between HFNC and COT groups [23, 24].

Recently, some meta-analyses tried to assess the efficiency of HFNC therapy in ARF patients. However, there were controversial results between these studies. [25, 26] After fully reviewing these meta-analyses, we found some studies that evaluated HFNC therapy in post-extubation patients were also involved in these studies. Though some post-extubation patients may suffer reintubation due to ARF, they constitute a different patient population rather than actual ARF patients. In the present systematic review and meta-analysis, we sought to evaluate whether there were differences between HFNC therapy and COT in treating ARF patients rather than post-extubation patients regarding the escalation of respiratory support and other aspects.

## Methods

We performed this systematic review and meta-analysis according to the guidelines described in the Cochrane Handbook for Systematic Reviews of Interventions [27] and PRISMA statements.

### Study selection criteria

#### Types of studies

Randomized controlled trials (RCTs) comparing HFNC therapy and COT in the treatment of ARF patients were included. As described previously, RCTs comparing HFNC therapy and COT in post-extubation patients were excluded.

#### Types of participants

Adult patients who had ARF, as defined by the authors of each study, were included.

#### Types of interventions

Trials comparing HFNC therapy with COT were eligible.

The intervention for the HFNC group was oxygen therapy provided through HFNCs and the control group received COT through nasal prongs, facemasks or Venturi masks. In addition, NIV was not included in the COT group in the present meta-analysis.

#### Types of outcome measures

Our primary outcome was the rate of escalation of respiratory support and the secondary outcomes included the following variables: intubation rate, mortality at the longest study follow-up, transfers to the ICU and complications.

### Data sources and search strategy

We searched for relevant studies published in the EMBASE, Medline, and Wanfang databases and the Cochrane Library.We also reviewed the references of relevant articles to avoid a loss of studies. We searched all relevant articles published from inception to June 2016. We used the following keywords and Emtree and MeSH terms in different combinations for the searches: “oxygen therapy”, “Oxygen inhalation therapy”, “Oxygen delivery devices”, “standard oxygen”, “high flow nasal cannula”, “high flow oxygen therapy”, “nasal high flow oxygen therapy”, “Nasal Cannula”, “acute respiratory failure”, and “respiratory failure”. No limits on the location of the trial, gender, age, sample size, or language were entered for the search. The full search strategies are shown in Additional file 1.

### Data extraction and quality assessment

Two investigators independently screened the titles and abstracts using a standardized data extraction form. Disagreements were resolved by consensus or by consulting a third author. We extracted the following data: authors’ names, the title of the article, the year and country of the study, the journal in which the study was published, laboratory results and clinical outcomes. The modified Jadad score was used to assess the quality of the included studies. Two independent investigators evaluated the risk of bias of the included studies according to the methods described in the Cochrane Handbook [25]. Studies were assessed according to the following domains: participant and personnel blinding, random sequence generation, allocation concealment, incomplete outcome data, blinding for outcome assessments, selective outcome reporting and other sources of bias. According to the Cochrane Handbook, other sources of bias were related to the specific study design or to the early termination of the involved trials because of extreme baseline imbalances in the selected samples. Blinding could not be implemented due to the nature of these studies.

### Statistical analysis

We used Review Manager Software 5.3 (RevMan 5.3, The Cochrane Collaboration, Oxford, United Kingdom) for the meta-analysis. Data were obtained by direct extraction or by indirect calculation. Binary data such as the rate of escalation of respiratory support and the intubation rate were expressed as risk ratios (RRs) and 95% confidence intervals (CIs). Heterogeneity between the studies was evaluated using the chi-square test and *P* < 0.05 with *I* ^2^ greater than 50% indicated significant heterogeneity. A fixed effects model and a random effects model were used in the absence and presence of statistical heterogeneity, respectively. The results were graphically displayed using forest plots and the potential publication bias was analyzed by visual inspection of the funnel plot.

Because the durations of HFNC treatment were different in each study, we conducted a subgroup analysis according to the duration of HFNC therapy (< 24 h v. ≥24 h).

### Sensitivity analysis

To test the reliability of the results, sensitivity analyses were also performed by repeating the present meta-analysis after removing one RCT at a time.

## Results

**Fig. 1:**
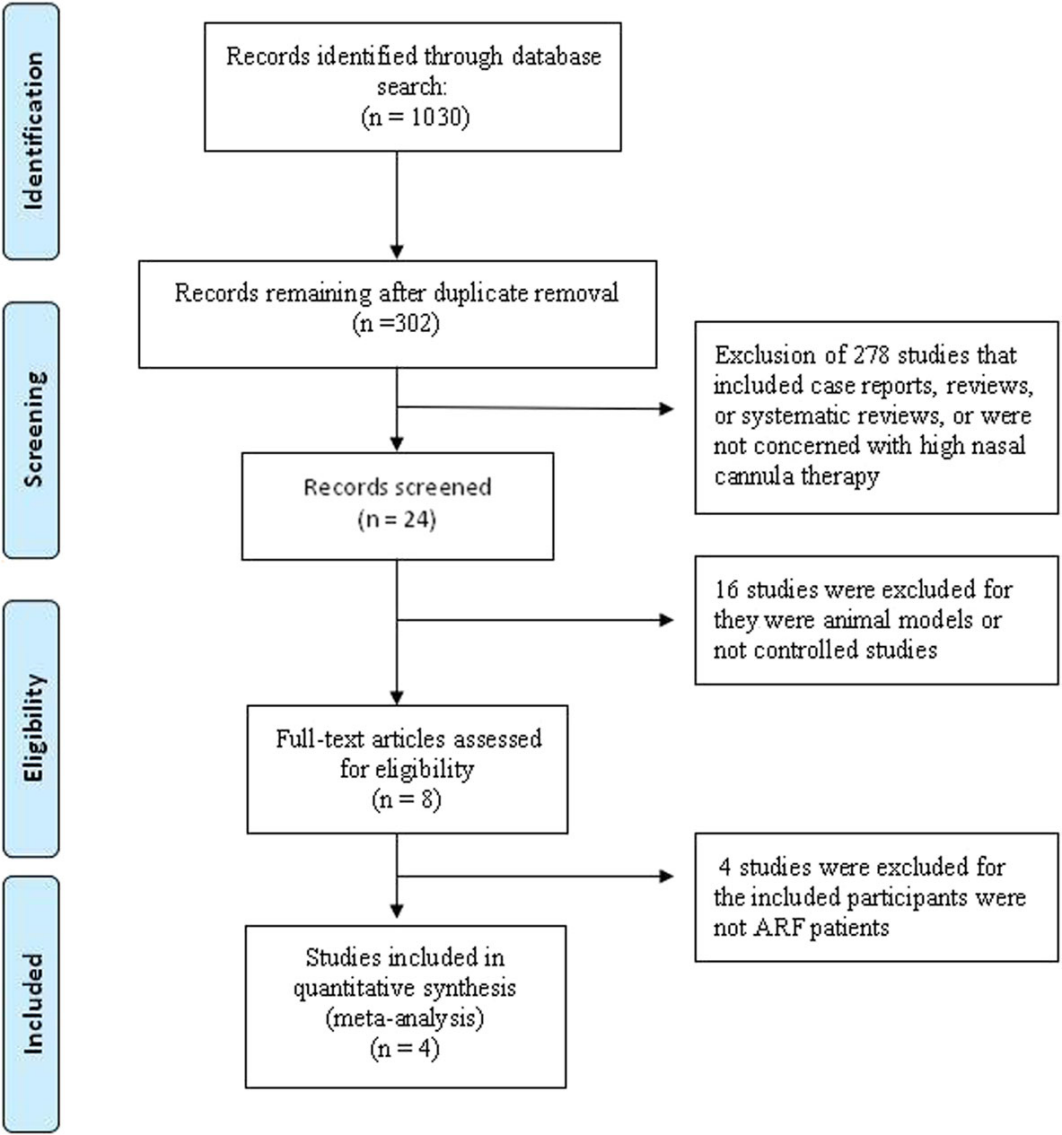
PRISMA flow diagram of the study selection process

The selection process of the eligible studies is shown in Fig. 1. Initially, 1030 potentially relevant records were identified. By screening the titles and evaluating the abstracts, we removed duplicate studies, reviews, case reports, animal studies, comments, and studies that were not randomized controlled studies, resulting in 8 studies that remained for assessment. Of these, 1 study compared HFNC therapy with NIV [28], 1 study evaluated the effect of HFNC therapy during endotracheal intubation [29], and 2 studies were conducted to prevent ARF after planned extubation [12, 30]. These studies were excluded. Finally, 4 studies were included in the present meta-analysis [2, 22–24]. The quality of the included studies is shown in Table 1.

**Table 1:** Quality of the included studies

Study	Randomization method	Blind method	Allocation concealment	Withdrawals/Dropouts (NG/NJ)	Jadad score
Bell 2015	Computer-generated random numbers	Not used	An opaque, sealed envelope system	Yes	5
Frat 2015	Permuted-block randomization	Not used	Centralized web based management system	Yes	5
Lemiale 2015	Permuted-block randomization	Not used	An opaque, sealed envelope system	Yes	5
Jones 2016	Computer-generated random numbers	Not used	An opaque, sealed envelope system	Yes	5

The modified Jadad score was used to evaluate the quality of the included trials

A total of 703 patients with ARF were included in this meta-analysis. Of these patients, 371 were randomly assigned to the HFNC group and 332 were assigned to the COT group. Table 2 shows the basic demographic characteristics of all included patients.

**Table 2:** Basic demographic parameters of patients in the included studies

Study	n	Age (years)	Gender (M/F)	Patients	Duration of HFNC or COT	Starting flow of HFNC	RRs (breaths/min)	P/F (mmHg)
Bell 2015								
HFNC group	48	72.9 ± 15.1	20/28	Emergency patients with ARF	2 h	50 L/min	>25	Unknown
COT group	52	74.5 ± 14.0	24/28		2 h			
Frat 2015								
HFNC group	106	61 ± 16	75/31	ICU patients with hypoxemic ARF	At least 48 h	50 L/min	>25	≤300
COT group	94	59 ± 17	63/31		At least 48 h			
Lemiale 2015								
HFNC group	52	59.3(43-70)*	38/14	Immunocompromised ICU patients with hypoxemic ARF	2 h	40-50 L/min	>30	Unknown
COT group	48	64.5(53.25-72)*	32/16		2 h			
Jones 2016								
HFNC group	165	74.6 ± 15.6	73/94	Emergency patients with ARF	24 h	40 L/min	≥22	Unknown
COT group	138	72.2 ± 16.8	71/67		24 h			

Plus–minus values are means ± SD; * values are median (25th–75th percentile); *M* male, *F* female. *ARF* acute respiratory failure, *HFNC* high flow nasal cannula, *COT* conventional oxygen therapy, *L/min* liter per minute, *RRs* respiratory rates,*P/F* PaO_2_/FiO_2_

### Risk of bias in the included studies

**Fig. 2:**
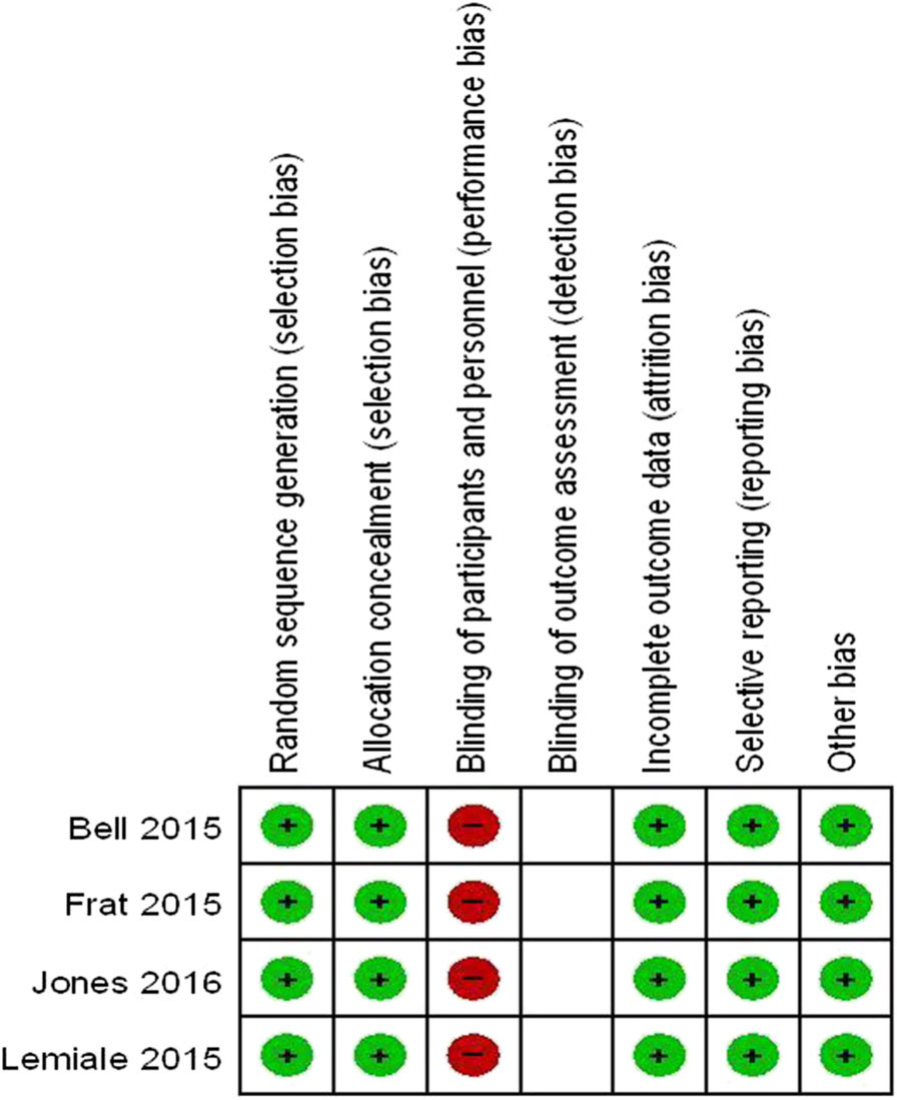
Risk of bias summary: the reviewers’ judgments about each risk of bias item for each included study

The risk of bias of each study was evaluated according to the methods described in the Cochrane Handbook and the details of the results are presented in Fig. 2.

### Escalation of respiratory support

**Fig. 3:**
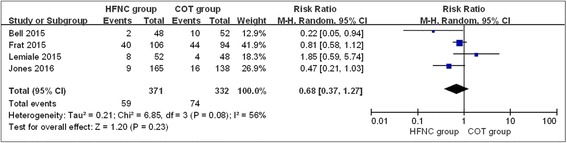
Escalation of respiratory support in the HFNC and COT groups

When initial treatment (HFNC therapy or COT) failed, an escalation of respiratory support was needed. All the included studies reported the rate of escalation of respiratory support. The strategies for the escalation of respiratory support in the 4 included studies were different (Table 3). There was significant heterogeneity between the studies (chi^2^ = 6.85, df = 3, *P* = 0.08; *I* ^2^ = 56%). In the random-effects model, the HFNC group did not show a significant difference compared with the COT group (RR, 0.68; 95% CI, 0.37, 1.27; z = 1.20, *P* = 0.23, Fig. 3).

**Table 3:** Strategies for escalation of respiratory support among included studies

Study	COT group	HFNC group
Bell 2015	HFNC, Noninvasive or invasive ventilation	Noninvasive or invasive ventilation
Frat 2015	Invasive ventilation	Invasive ventilation
Lemiale 2015	Noninvasive or invasive ventilation	Noninvasive or invasive ventilation
Jones 2016	Noninvasive or invasive ventilation	Noninvasive or invasive ventilation

*COT* conventional oxygen therapy, *HFNC* high flow nasal cannula oxygen therapy

**Fig. 4:**
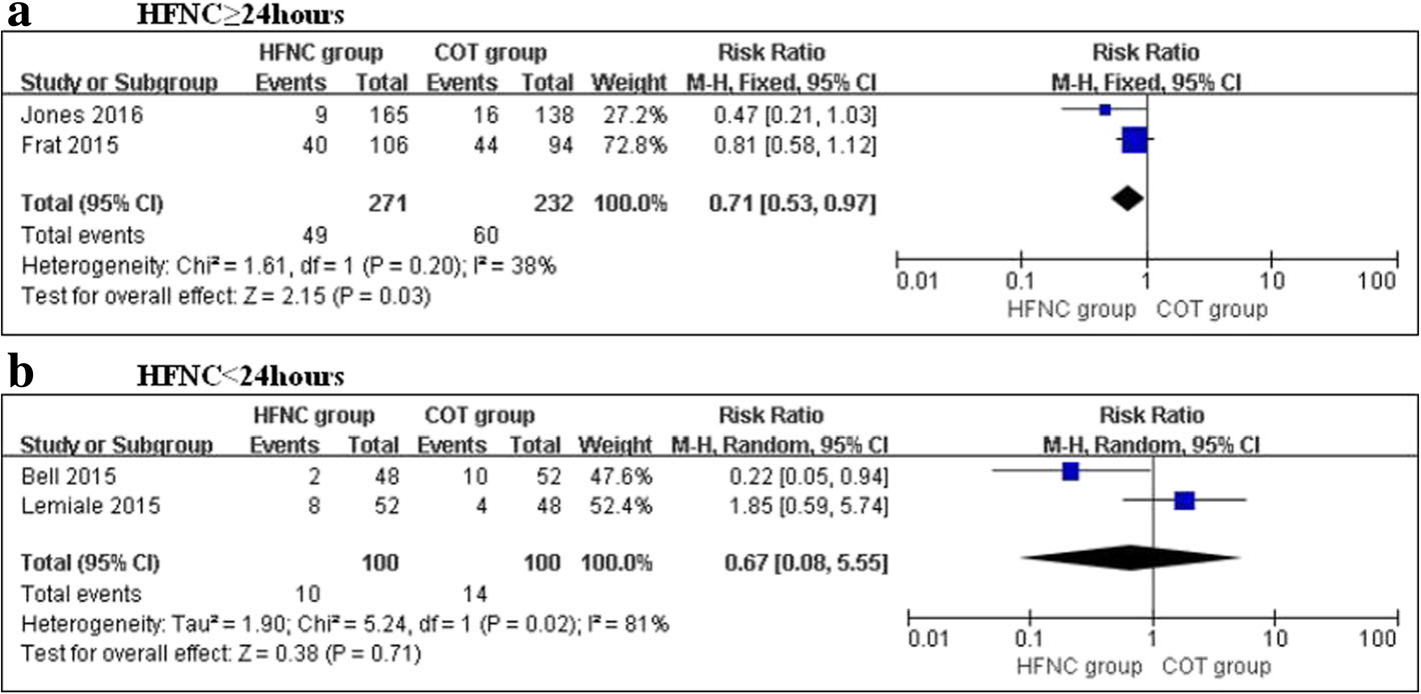
Subgroup analysis of escalation for respiratory support in the HFNC and COT groups: (**a**) HFNC ≥ 24hours; (**b**) HFNC < 24hours

The subgroup analysis showed a significant 29% decrease in the escalation of respiratory support in the HFNC group when the patients were treated with HFNC therapy for ≥24 h compared with COT (RR, 0.71; 95% CI, 0.53, 0.97; z = 2.15, *P* = 0.03, Fig. 4). HFNC therapy did not demonstrate any benefit over COT in patients treated for less than 24 h (RR, 0.67; 95% CI, 0.08, 5.55; z = 0.38, *P* = 0.71, Fig. 4).

### Intubation rate

**Fig. 5:**
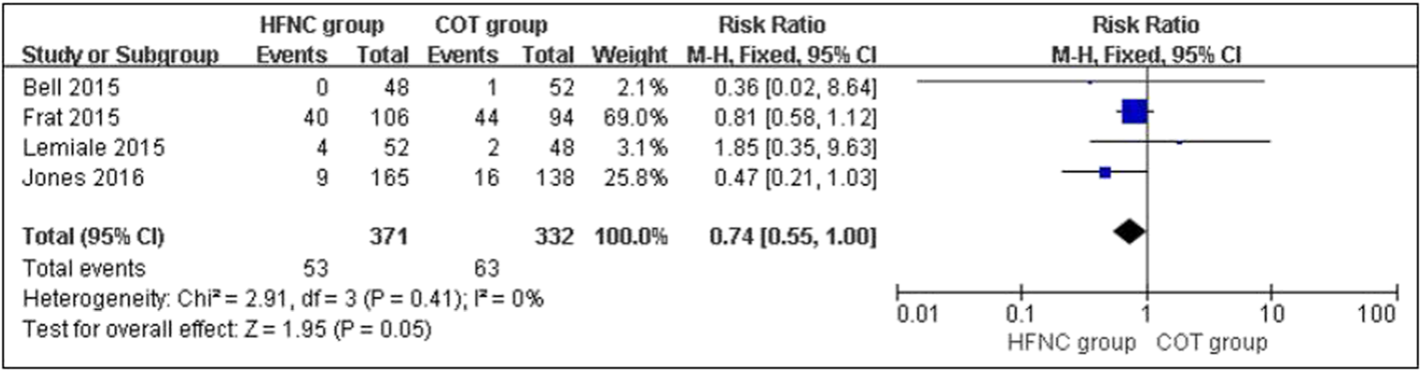
Intubation rates in the HFNC and COT groups

All the included studies reported intubation rates. When the results of the 4 studies were analyzed, no significant heterogeneity was observed between the studies (chi^2^ = 2.07, df = 3, *P* = 0.56; *I* ^2^ = 0%). The intubation rates of the COT group and the HFNC group were similar, with no significant difference between the two groups (RR, 0.74; 95% CI, 0.55, 1.00; z = 1.95, *P* = 0.05, Fig. 5).

**Fig. 6:**
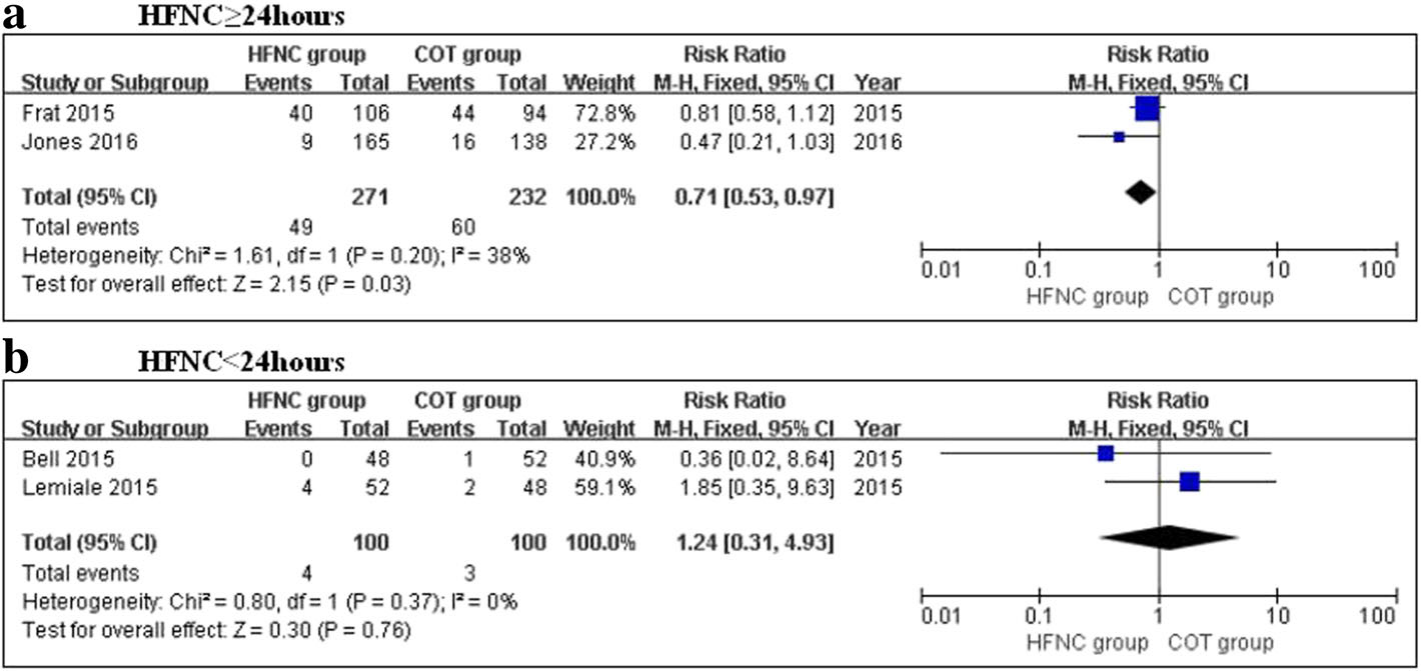
Subgroup analysis of intubation rate in the HFNC and COT groups: (**a**) HFNC ≥ 24hours; (**b**) HFNC < 24hours

The subgroup analysis also shown a significant decrease in the intubation rate in the HFNC group when patients were treated with HFNC therapy for ≥24 h compared with COT (RR, 0.71; 95% CI, 0.53, 0.97; z = 2.15, *P* = 0.03, Fig. 6). HFNC therapy did not demonstrate any benefit over COT in patients treated for less than 24 h (RR, 1.24; 95% CI, 0.31, 4.93; z = 0.30, *P* = 0.76, Fig. 6).

### Mortality

**Fig. 7:**
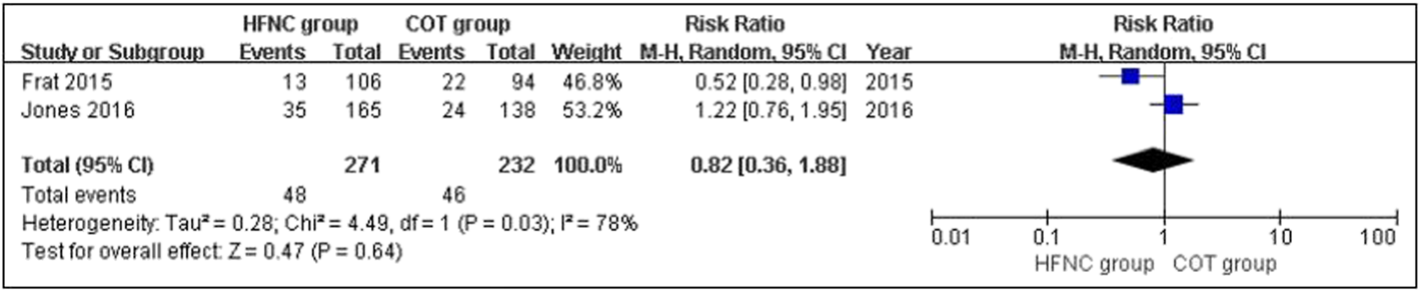
Mortality between the HFNC and COT groups

Two of the four included studies reported mortality [22, 24]. There was significant heterogeneity between the studies (chi^2^ = 4.49, df = 1, *P* = 0.03; *I* ^2^ = 78%). In addition, HFNC oxygen therapy did not decrease mortality compared with COT (RR, 0.82; 95% CI, 0.36, 1.88; z = 0.47, *P* = 0.64, Fig. 7).

### Rate of transfer to the ICU

**Fig. 8:**
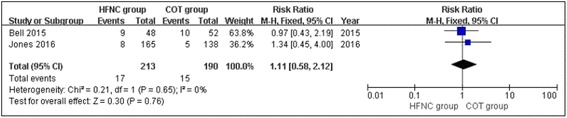
Rate of transfers to the ICU between the HFNC and COT groups

Two of the four studies were conducted in the emergency department and both reported the rate of admission to the ICU [2, 24]. No significant heterogeneity was observed between the two studies (chi^2^ = 0.21, df = 1, *P* = 0.65; *I* ^2^ = 0%) and there was no significant difference in the rate of ICU transfer between the two groups (RR, 1.09; 95% CI, 0.57, 2.09; z = 0.26, *P* = 0.79, Fig. 8).

### Complications

Since the data on complications were insufficiently reported in the 4 included studies, we can only provide a description of their occurrence. In the studies by Bell et al. and Lemiale et al., no severe complications (i.e., nasal mucosa, skin trauma, infectious complications or hemodynamic instability due to HFNC) were reported. In the studies by Frat et al. and Jones et al., the overall incidence of serious adverse events was similar in the HFNC and COT groups (data not reported).

**Fig. 9:**
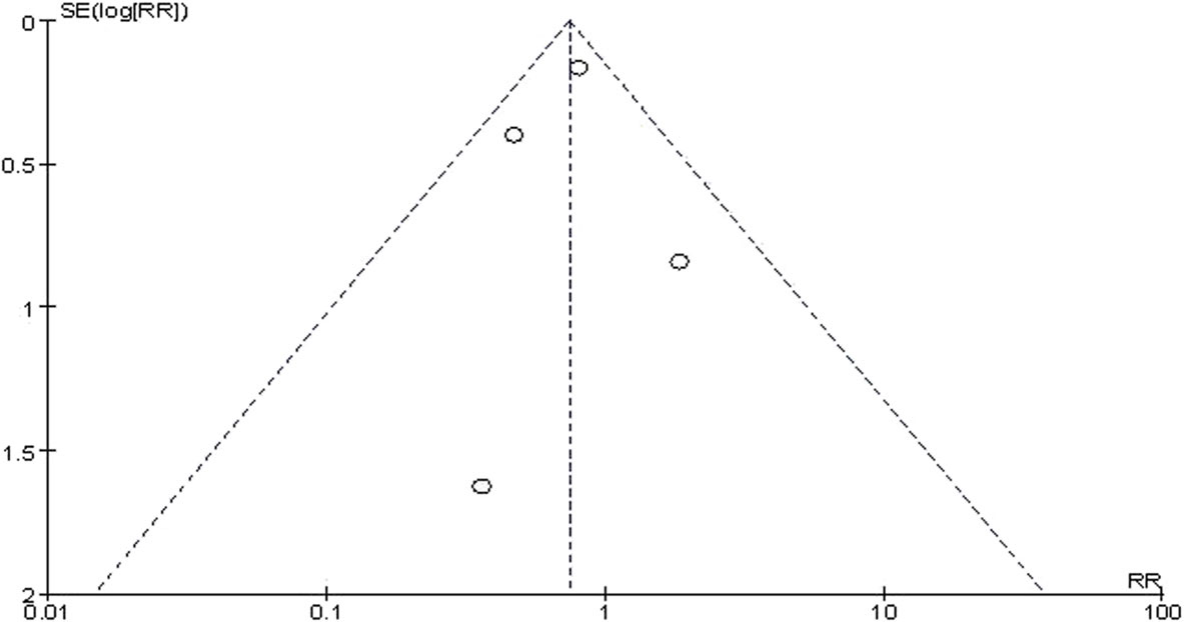
Funnel plot for publication bias

No publication bias was observed based on a visual inspection of the funnel plot (Fig. 9).

We had planned to analyze other variables, such as the lengths of stay in the ICU or hospital, patient comfort, the duration of HFNC therapy, maximal flow of the HFNC, and the costs of the two methods, but these variables were either not researched or were insufficiently reported in the included trials. Therefore, we could not perform any analyses with regard to these data.

## Discussion

Recently, HFNC oxygen therapy has achieved widespread use in adult ARF patients in emergency departments and intensive care units [20, 31, 32]. However, the effect of HFNC therapy in adult ARF patients remains inconclusive. The present meta-analysis included 4 RCTs that studied 703 patients (371 HFNC and 332 COT patients) to examine whether there were differences between HFNC therapy and COT in the treatment of ARF. The overall estimates of this meta-analysis showed that there were no significant differences between the HFNC and COT groups in the rates of escalation of respiratory support (RR, 0.68; 95% CI, 0.37, 1.27; z = 1.20, *P* = 0.23), intubation (RR, 0.74; 95% CI, 0.55, 1.00; z = 1.95, *P* = 0.05), mortality (RR, 0.82; 95% CI, 0.36, 1.88; z = 0.47, *P* = 0.64), or ICU transfer (RR, 1.09; 95% CI, 0.57, 2.09; z = 0.26, *P* = 0.79) in the treatment of ARF. Our results were similar to a previous study [26].

Although the present meta-analysis found no significant differences between HFNC therapy and COT for the treatment of adult ARF patients, it should be noted that there was significant heterogeneity between the RCTs included in the present study, which may have affected our conclusions. A series of factors may have led to this significant heterogeneity. First, as shown in Table 4, the criteria for ARF differed between the 4 studies (Table 4). The different criteria for inclusion led to different degrees of ARF among the patients in the four studies. In the study by Frat and colleagues, there was no significant difference in the intubation rate between the two groups, but when they conducted a post hoc analysis according to the ratio of PaO_2_/FiO_2_ at enrollment (≤ 200 mmHg versus >200 mmHg), they found that for the subgroup of patients with a PaO_2_/FiO_2_ ≤ 200 mmHg, the intubation rate was significantly lower in the HFNC group than in the COT group [22]. Therefore, we consider the degree of ARF to be an important factor influencing the effectiveness of HFNC therapy. In addition, the differences in the degree of ARF experienced by the patients in the present meta-analysis may have eliminated the potential differences between the two groups. In fact, some therapies may only be useful in more critically ill patients.

**Table 4:** The criteria for ARF among the included studies

Bell 2016	Frat 2015	Lemiale 2015	Jones 2015
1. RRs >25 breaths /min 2. SpO_2_ < 93%	1. RRs >25 breaths/min 2. PaO_2_ / FiO_2_ ≤ 300 mmHg when the patients breathed oxygen at a flow rate > 10 l/min over 15 min 3. PaCO_2_ ≤ 45 mmHg 4. An absence of clinical history of underlying chronic respiratory failure	1. A need for oxygen greater than 6 L/min to maintain SpO_2_ > 95% 2. Symptoms of respiratory distress*	1. SpO_2_ ≤ 92% on air 2. RRs ≥22 breaths/min

*RRs* respiratory rates, *SpO*_*2*_ peripheral capillary oxygen saturation, *PaO*_*2*_ arterial partial pressure of oxygen, *FiO*_*2*_ fraction of the inspired oxygen, *PaCO*_*2*_ partial pressure of arterial carbon dioxide, *Min* minute; *: tachypnea >30/min, intercostal recession, labored breathing, and/or dyspnea at rest

Second, the starting flow of HFNC therapy may also affect results. The starting flows of HFNC therapy differed between the four studies (Table 2). In a study by Parke et al., researchers measured patients’ nasopharyngeal pressure when HFNC therapy was performed with gas flows of 30, 40, and 50 L/min [14]. They found that during HFNC therapy, the mean nasopharyngeal airway pressures were 1.5 ± 0.6, 2.2 ± 0.8, and 3.1 ± 1.2 mmHg at 30, 40, and 50 L/min, respectively. They demonstrated that the level of PEEP as a benefit of HFNC therapy was flow-dependent. The different starting flows may have led to different levels of PEEP and could have affected the results. Consistently, in two studies by Bell et al. and Frat et al., the starting flows were all 50 L/min and these studies showed more benefits in the HFNC group than in the COT group. [2, 22]

Third, as shown in Table 3, the criteria for the escalation of respiratory support differed between the 4 studies. In addition, the strategies for escalation also differed. The different criteria and strategies used for the escalation of respiratory support may have led to a bias. Furthermore, three of the studies included the option of escalating to NIV as a strategy for the escalation of respiratory support in the HFNC group. However, whether the patients who failed to improve with HFNC therapy could be recovered by escalating to NIV is currently unclear [8].

The subgroup analysis showed that HFNC therapy may decrease the rates of escalation of respiratory support (RR, 0.71; 95% CI, 0.53, 0.97; z = 2.15, *P* = 0.03) and intubation (RR, 0.71; 95% CI, 0.53, 0.97; z = 2.15, P = 0.03) when ARF patients are treated with HFNC therapy for ≥24 h compared with COT, which is not surprising. As we know, some therapies may only be useful with a sufficient duration. In the other two RCTs [2, 23], the durations of HFNC therapy were only 2 h, which may be too short to show its benefit. HFNCs have shown greater benefits with longer durations in post-extubation patients. A previous randomized controlled study by Maggiore et al. compared HFNC therapy with COT in 105 patients after extubation [12]. The duration of HFNC therapy was at least 48 h. Notably, the results showed that the reintubation rate or any form of escalation of respiratory support was significantly lower in the HFNC group than in the COT group. The duration of HFNC therapy in their study was similar to that used in the study by Frat et al., which was included in this meta-analysis. In addition, in the study by Frat et al., HFNC oxygen therapy also showed a positive effect in decreasing 90-day mortality [22]. Conversely, when the duration of HFNC oxygen therapy was shorter, studies comparing HFNC therapy and COT often showed negative results, including the study by Lemiale et al. that was included in the present meta-analysis [23]. Therefore, it seems that the duration of HFNC therapy is associated with its efficacy, with longer durations of HFNC therapy potentially leading to better results. The optimal duration of HFNC therapy in patients with ARF is still unclear and the durations of HFNC therapy in the relevant studies varied greatly. Our present meta-analysis could provide useful information in this regard.

There are several limitations of our meta-analysis. First, there were few studies that compared HFNC therapy and COT in patients with ARF and the number of patients included in our meta-analysis was limited. According to the study by Frat et al., the intubation rate was 38% in the HFNC group and 47% in the COT group. Assuming an intubation rate of 45% in the COT group, to detect a 5-percentage point reduction in the intubation rate in the HFNC group with an α of 0.05 and a β of 0.20 would require each group to enroll approximately 1220 subjects. Therefore, further large-scale studies are needed to confirm our results. Second, the effects of HFNC therapy in hypoxic ARF may be different from those in hypercapnic ARF, however, we could not perform a subgroup analysis relative to this aspect due to a lack of raw data. Third, since only 4 studies were included in the present meta-analysis, a funnel plot could not provide sufficient power to reveal a publication bias. Fourth, it should be noted that the durations of HFNC therapy were different among the 4 studies, especially in the studies by Bell and Lemiale [2, 23] in which the durations of HFNC therapy were only 2 h. Our subgroup analysis showed that a longer duration of HFNC therapy (≥24 h) may benefit ARF patients, so the inclusion of studies with different durations of HFNC therapy might produce biases.

## Conclusions

Our meta-analysis demonstrated that HFNC therapy was similar to COT in ARF patients. The subgroup analysis showed that HFNC therapy may decrease the rates of the escalation of respiratory support and intubation when ARF patients were treated with HFNC therapy for ≥24 h compared with COT. Further high-quality, large-scale studies are needed to confirm our results.

## Additional file

Additional file 1: Details of Search Strategy. (DOC 28 kb)

## Abbreviations

ARF: Acute respiratory failure
CIs: Confidence intervals
COT: Conventional oxygen therapy
F: Female
FiO_2_: Fraction of inspired oxygen
HFNC: High-flow nasal cannula
IMV: Invasive mechanical ventilation
M: Male
NIV: Noninvasive ventilation
RCTs: Randomized controlled trials
RRs: Risk ratios
