# Supplementary material for: High-flow nasal cannula oxygen therapy versus conventional oxygen therapy in patients with acute respiratory failure: a systematic review and meta-analysis of randomized controlled trials
Source: BMC Pulm Med. 2017 Dec 13;17:201. doi: 10.1186/s12890-017-0525-0 (PMC5729290; doi:10.1186/s12890-017-0525-0)
Supplement: Additional file 1: — Details of Search Strategy. (DOC 28 kb) [file 12890_2017_525_MOESM1_ESM.doc]

**Additional file 1. Details of Search Strategy**

1. **Medline**
   1. oxygen therapy. ti,ab,kw.
   2. oxygen inhalation therapy. ti,ab,kw.
   3. oxygen delivery devices. ti,ab,kw.
   4. standard oxygen. ti,ab,kw.
   5. high flow nasal cannula. ti,ab,kw.
   6. high flow oxygen therapy. ti,ab,kw.
   7. nasal high flow oxygen therapy. ti,ab,kw.
   8. nasal cannula. ti,ab,kw.
   9. 1 or 2 or 3 or 4 or 5 or 6 or 7 or 8
   10. respiratory failure.ti,ab,kw.
   11. acute respiratory failure.ti,ab,kw.
   12. 10 or 11
   13. 9 and 12
   14. random.ti,ab,kw.
   15. randomly.ti,ab,kw.
   16. randomized.ti,ab,kw.
   17. 16 or 17 or 18
   18. 13 and 17
2. **Embase**
   1. oxygen therapy. ti,ab,kw.
   2. Oxygen inhalation therapy. ti,ab,kw.
   3. Oxygen delivery devices. ti,ab,kw.
   4. standard oxygen. ti,ab,kw.
   5. high flow nasal cannula. ti,ab,kw.
   6. high flow oxygen therapy. ti,ab,kw.
   7. nasal high flow oxygen therapy. ti,ab,kw.
   8. Nasal Cannula. ti,ab,kw.
   9. 1 or 2 or 3 or 4 or 5 or 6 or 7 or 8
   10. respiratory failure.ti,ab,kw.
   11. acute respiratory failure.ti,ab,kw.
   12. 10 or 11
   13. 9 and 12
   14. random.ti,ab,kw.
   15. randomized.ti,ab,kw.
   16. 14 or 15
   17. 13 and 16
3. **Cochrane Library**
   1. oxygen therapy. ti,ab,kw.
   2. Oxygen inhalation therapy. ti,ab,kw.
   3. Oxygen delivery. ti,ab,kw.
   4. standard oxygen therapy. ti,ab,kw.
   5. high flow nasal cannula. ti,ab,kw.
   6. high flow oxygen therapy. ti,ab,kw.
   7. nasal high flow oxygen therapy. ti,ab,kw.
   8. Nasal Cannula. ti,ab,kw.
   9. 1 or 2 or 3 or 4 or 5 or 6 or 7 or 8
   10. respiratory failure.ti,ab,kw.
   11. 9 and 10
   12. random.ti,ab,kw.
   13. randomly.ti,ab,kw.
   14. randomized.ti,ab,kw.
   15. 12 or 13 or 14
   16. 11 and 15
4. **Wanfang Database**

主题:(经鼻高流量吸氧) or 主题:(鼻导管吸氧) or 主题:(氧疗) or 主题:(标准氧疗) or 主题:(标准氧疗) + 主题:(呼吸衰竭)
